# Amphibian Intestine Allometry

**DOI:** 10.1002/jmor.70130

**Published:** 2026-05-19

**Authors:** M. J. Duque‐Correa, C. Meloro, S. Keller, P. Cigler, I. Wethli, J. Niehaus, M. Przybyło, M. Clauss

**Affiliations:** ^1^ Clinic for Zoo Animals, Exotic Pets and Wildlife, Vetsuisse Faculty, University of Zurich Zurich Switzerland; ^2^ Research Center in Evolutionary Anthropology and Palaeoecology, School of Biological and Environmental Sciences, Liverpool John Moores University Liverpool UK; ^3^ Institute for Fish and Wildlife Health, Department of Infectious Diseases and Pathobiology, Vetsuisse Faculty University of Bern Bern Switzerland; ^4^ Department of Animal Nutrition and Fisheries University of Agriculture in Krakow Krakow Poland

**Keywords:** anatomy, digestion, scaling

## Abstract

Across four large vertebrate groups—fish, reptiles, birds and mammals— intestine length has been shown to scale hyper‐allometrically with body mass (BM), at an exponent higher than the geometric (isometric) expectation, 0.33. So far, amphibians have not been investigated in this respect. Combining original data from dissections and literature data, we evaluated the scaling of total intestine length with BM in the adult stages of 38 amphibian species (37 of which anurans). The BM range of investigated taxa was 2.2 to 113.5 g. When accounting for phylogeny, intestine length scaled with BM at an exponent with a 95% confidence interval of 0.39 to 0.53, corroborating the hyper‐allometric scaling observed in other vertebrates. The hypothetical explanation is that larger animals, while requiring a proportionate intestinal absorptive surface to meet their metabolic demands, need to maintain short diffusion distances between the sites of digestive enzyme secretion/nutrient absorption and the digesta. This mechanism should result in hypo‐allometric scaling of intestine diameter, with the hyper‐allometric scaling of intestinal length ensuring the overall constancy of functional organ surface.

## Graphical abstract

Total intestine length scales hyper‐allometrically, that is, at an exponent > 0.33, with body mass across vertebrates, including amphibians.

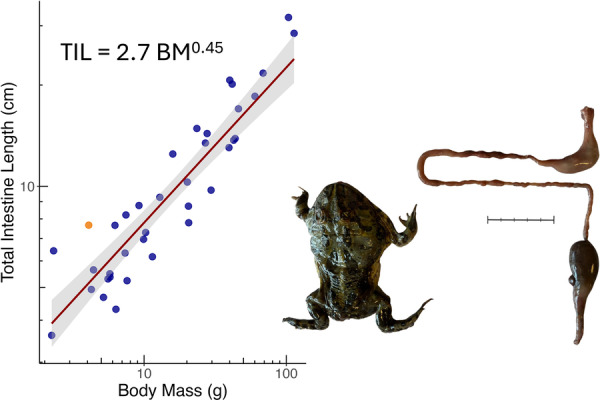

## 1 Introduction

In studies on the digestive tract of vertebrates, the length of the intestinal tract is one of the few quantitative measures that lends itself to large‐scale comparisons across taxa, as done in mammals (Duque‐Correa et al. 2021), reptiles (Hoppe et al. 2021), birds (Duque‐Correa et al. 2022), and fishes (Duque‐Correa et al. 2024). Intestinal length can also be correlated quantitatively with measures of digestive physiology (Duque‐Correa et al. 2025), or with other anatomical measurements such as brain size in the framework of the expensive tissue hypothesis (Liao et al. 2016). Two fundamental issues are typically targeted in such studies: on the one hand, relationships with other biological characteristics of species—mainly, but not exclusively, their natural diet; on the other hand, the scaling of intestine length with body size.

The scaling of intestine length with body mass (BM) is noteworthy because generally, it deviates from the expected geometric (isometric) scaling exponent of 0.33; a length should scale to a volume or a mass at an exponent of 0.33 (e.g., Calder 1996). However, deviation from this pattern is the norm across vertebrates, yielding higher scaling exponents in a “hyper‐allometric” scaling for intestine lengths. This was described in various data collections within mammals and birds (Lavin et al. 2008, Duque‐Correa et al. 2021, Duque‐Correa et al. 2022), reptiles (Hoppe et al. 2021), and fishes (Duque‐Correa et al. 2024), and in more limited collections on mammalian carnivores (McGrosky et al. 2016), and ruminants (Woodall and Skinner 1993; McGrosky et al. 2019a). Within mammals, the main exceptions are rodents and primates, for which scaling exponents similar to the geometric expectation have been described (Lovegrove 2010; McGrosky et al. 2019b), but a larger data collection indicated hyper‐allometric or close‐to‐hyper‐allometric scaling (at an exponent > 0.33) in these taxa as well (Duque‐Correa et al. 2021). In birds, hyper‐allometric scaling was observed in passerines (Herrera 1986) and seabirds (Jackson 1992). The general explanation is that for optimal digestion, diameters of intestines should not increase isometrically with BM as diffusion distances would become too long. However, the total intestinal surface has to support the metabolism of the animal. Due to the similarity between geometric surface scaling (at a body mass exponent of 0.67, Calder 1996) and the general scaling of metabolism (at body mass exponents around 0.67–0.75, e.g., Glazier 2005), the basic expectation is that intestinal surface should scale as expected by metabolism and geometry. This was corroborated by Karasov and Hume (1997) who showed that, within different vertebrate clades, the scaling of intestinal surface with body mass had an exponent of 0.71. Hence, as the intestinal surface is expected to scale geometrically, but intestinal diameter should have a lower scaling, length must necessarily scale hyper‐allometrically to compensate (Woodall and Skinner 1993).

In compiling data sets on vertebrate taxa for the studies cited above, we realized that data for the intestine length of Amphibia are surprisingly scarce in the literature. The digestive tract of adult Amphibia may not have been a focus of comparative research because they are usually considered homogenous in terms of their faunivorous diet; the consistent ingestion of plant material by adult amphibia has only been reported in a few species, including sirenid salamanders (Hill et al. 2015), *Xenohyla truncata* (de‐Oliveira‐Nogueira et al. 2023), *Rhinella marina* (Isaacs and Hoyos 2010), and *Rana hexadactyla* (Das 1996). With respect to sirenids, Pryor et al. (2006) reported that their gastrointestinal tract did not show the adaptations to herbivory typically observed in other vertebrates. Thus, the amphibian gastrointestinal tract may not have held much interest for researchers. To our knowledge, the main exceptions were when comparing principles of intestinal digestion between vertebrate classes (Buddington et al. 1991), corroborating that intestinal surface differs across vertebrates in correspondence with metabolic requirements (Karasov and Hume 1997), and in connection with a tradeoff with other organs in the framework of the expensive tissue hypothesis (Liao et al. 2016).

Therefore, we also did not expect distinct variation in the intestinal length of Amphibia in relation to their trophic behavior; however, we aimed to assess whether the scaling of intestinal length with BM is hyper‐allometric in Amphibia as it is in other vertebrates. For this aim, we obtained data on the length of the intestinal tract of 37 anuran and one caudate Amphibia, and analyzed the relationships of BM, body length (BL), and intestine length while accounting for the phylogenetic relationships between species.

## 2 Materials and Methods

The data set for this study originated from available published data and additional unpublished data from post‐mortem examinations. The published data were collected following a thorough literature search. Publications on amphibian intestinal length were searched using Google Scholar. Search terms included “anatomy”, “morphometry”, “digestive tract”, “intestine”, “gut”, and “length”, as well as taxon names. Additionally, the reference lists of identified sources and the references that cite them were scrutinized. Data from published sources were only included if the species, body mass (BM), and intestinal length were reported; if snout‐vent length (SVL) was reported, it was also included in the final data set. Only data for adult specimens were included. Information on the sex of these adults was missing in most cases. This yielded data for 32 species based on 401 individuals (Latimer and Roofe 1964; Toloza and Diamond 1990; Buddington et al. 1991; Naya et al. 2009; Liao et al. 2016; Yang et al. 2020; Mi and Liao 2021). To this collection, we added 22 individuals of six species by own dissections; these animals were local wildlife submitted for wildlife survey postmortem examinations (3 species) or deceased specimens of zoological institutions and private breeders (3 species). For a detailed list of species analyzed and their sources see the supporting material.

Before the dissections, the BM and SVL were measured. During dissections, the celomic cavity was accessed, and the gastrointestinal tract was removed from the cavity, the liver was separated, and the mesenteries were removed. The whole gastrointestinal tract was placed on millimeter paper without stretching and photographed. Total intestine length (TIL) was measured from the photographs using the ImageJ software (Schneider et al. 2012). TIL was measured from the stomach's pyloric sphincter to the anus.

The combination of own dissections and literature data meant that data derived from formalin‐fixated specimens (Liao et al. 2016) and non‐fixated specimens were combined. While studies using the same specimens—measuring dissected intestines before and after fixation—do not exist, to our knowledge, a previous study on intestine length in a primate species suggested no systematic difference in total intestinal length between fixated and non‐fixated specimens (Clauss et al. 2021). Therefore, combining these data was deemed acceptable.

A backbone phylogenetic tree containing all the selected species was identified by applying a maximum clade credibility tree extraction using the R package TreeTools (Smith 2019) from a subset of 1000 posterior phylogenies downloaded from vertlife.org and based on the Amphibia data set of Jetz and Pyron (2018). Clade frequencies were calculated across the posterior sample, and each tree was scored by summing the posterior support of its constituent clades. The tree with the highest cumulative clade support was selected as the MCC tree, providing a single representative ultrametric topology (with branch lengths as time of divergence in million years) for the comparative analyses (Heled and Bouckaert 2013).

The complete data set primarily consisted of anurans (37 species, 366 individuals), with only one species belonging to the order Caudata (the Eastern tiger salamander, *Ambystoma tigrinum*) (measurements from 57 individuals, reported as a species average) (Latimer and Roofe 1964); SVL was also available for 35 of these species. Data were aggregated into an average per species.

All statistical analyses were performed in R Studio, version 4.4.1 (R Core Team 2025) using generalized least squares (GLS) and phylogenetic generalized least squares (PGLS), recording the 95% confidence interval for parameter estimates, using the R packages “caper” (Orme et al. 2013) and “nlme” (Pinheiro et al. 2016). In all PGLS models, the parameter lambda (*λ*) was estimated by maximum likelihood. Analyses were performed on (i) all available data (*n* = 38 species), (ii) on a data set including all available anuran data (*n* = 37 species), and (iii) on a consistent data set, using only the species for which BM and SVL were available (*n* = 35 species). The significance level was set to 0.05. To compare models run for the same data subset, we used the small‐sample‐corrected Akaike information criterion (AICc), considering a difference between models when values are greater than 2 (ΔAICc > 2) (Burnham and Anderson 2002). Data visualization was done with the packages ggplot2 (Wickham 2016) and Patchwork (Pedersen 2019).

To explore the effect that body elongation has in amphibians, we included additional data on the greater siren (*Siren lacertina*) from Pryor et al. (2006). Data on the greater siren were not included in the main data set since it is based on total gut length, including the stomach, which would bias the results of statistical analyses. We only compared the two amphibian clades by calculating the scaling exponent of the relationship between intestine length, BM, and BL, and between BL and BM, as well as by visual assessment.

To visually compare amphibians with another aquatic vertebrate clade, we collected data on fish intestine length from (Duque‐Correa et al. 2024). We compared the length of the intestine relative to BM and BL between the two clades. SVL was used as a proxy for BL in amphibians, while standard length (SL) was the BL proxy for fishes. SVL and SL are considered somewhat comparable since they do not include the tail or fin; however, SL reaches beyond the vent (cloaca) that is the endpoint for the SVL measurement.

## 3 Results

**Figure 1:**
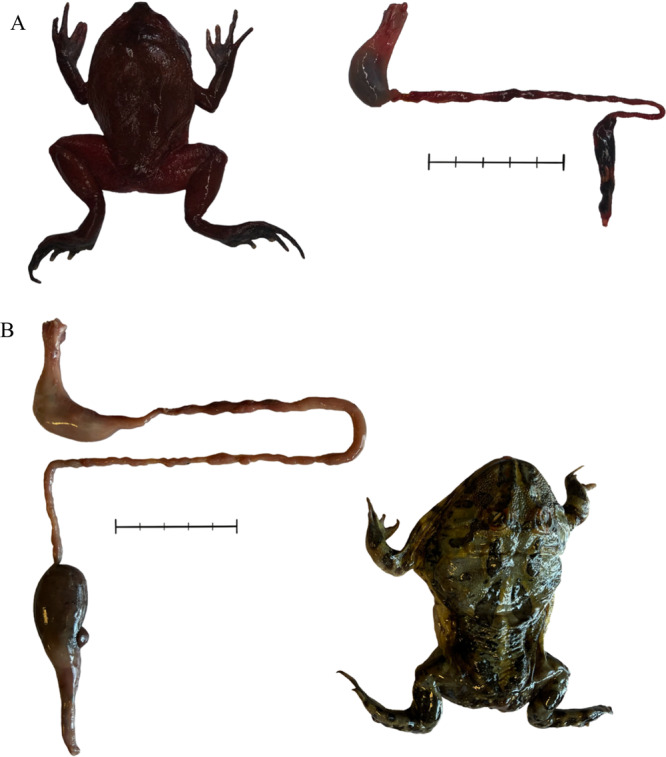
Gastrointestinal tract of (A) tomato frog, *Dyscophus antongilii*; (B) Cranwell's Horned Frog, *Ceratophrys cranwelli*. Bar = 5 cm.

The gastrointestinal tract of representative frogs was relatively short and simple, with a single‐chambered stomach followed by a short and narrow small intestine; there is no cecum, but a considerable dilation indicates the large intestine (Figure 1).

The phylogenetic signal lambda was significant for the relationship between BL and BM, suggesting taxon‐specific body shapes (Table 1). By contrast, it was not significant (95% CI including zero) for relationships of intestine length with either BM or BL.

**Table 1:** Complete data set: Summary statistics for allometric scaling as log(y) = a + b log, assessed with Generalized Least Squares (GLS) or – accounting for phylogenetic relationships – by Phylogenetic Generalized Least Squares (PGLS). *n* = number of species. Significant parameters in bold.

Dependent	Model	*n*		GLS	PGLS	parameter (95%CI)
				parameter (95%CI)	Lambda (95%CI)	
SVL	BM	35	a	**0.44 (0.37 to 0.51)**	0.98 (0.89 to NA)	**0.53 (0.42 to 0.65)**
			b	**0.26 (0.21 to 0.32)**		**0.28 (0.25 to 0.32)**
SVL	BM	34^a^	a	**0.39 (0.36 to 0.42)**	0.98 (0.89 to NA)	**0.39 (0.34 to 0.43)**
			b	**0.29 (0.27 to 0.32)**		**0.29 (0.27 to 0.32)**
Total intestine	BM	38	a	**0.43 (0.34 to 0.52)**	0.00 (NA to 0.629)	**0.43 (0.34 to 0.52)**
			b	**0.46 (0.39 to 0.53)**		**0.46 (0.39 to 0.53)**
Total intestine	SVL	35	a	0.01 (‐0.26 to 0.27)	0.69 (NA to 0.92)	−0.2 (‐0.51 to 0.19)
			b	**1.28 (0.93 to 1.63)**		**1.4 (1.01 to 1.73)**

Abbreviations: BM, body mass; SVL, snout‐vent length.

^a^ including only anurans.

**Figure 2:**
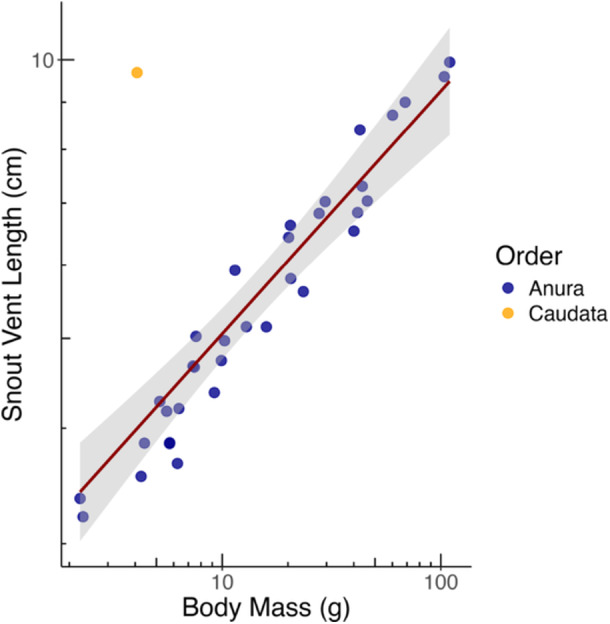
Snout‐vent length (SVL) relationship with body mass (BM) in amphibians, representing averages per species (*n* = 35 species). Regression line from GLS analysis; for statistics, see Table 1.

For amphibians, SVL scaled to BM at a nearly isometric exponent (with the 95%CI approaching 0.33) (Table 1 and Figure 2). The same was evident for the consistent data set (Table 2). The consistent data set included data on a Caudata species, the Eastern tiger salamander (*Ambystoma tigrinum*). When assessing the relationship between SVL and BM for all amphibians, the Eastern tiger salamander is a clear outlier, due to a more elongated shape compared to the anurans (Figure 2). When the salamander was removed from the analyses, data fit was improved as evident from smaller 95%CI, and the scaling exponent remained close to isometric (Table 1).

**Table 2:** Consistent data set: Summary statistics for allometric scaling as log(y) = a + b log, assessed with Generalized Least Squares (GLS) or – accounting for phylogenetic relationships – by Phylogenetic Generalized Least Squares (PGLS). *n* = 35 species. Significant parameters in bold.

Dependent	Model		GLS	ΔAICc	parameter (95%CI)	PGLS	AICc	ΔAICc	parameter (95%CI)
			AICc			Lambda (95%CI)			
SVL	BM	a	−64.6		**0.44 (0.37 to 0.51)**	0.98 (0.89 to NA)	−115.2		**0.53 (0.42 to 0.65)**
		b			**0.26 (0.21 to 0.32)**				**0.28 (0.25 to 0.32)**
Total intestine	BM	a	−43.0	0.0	**0.44 (0.34 to 0.53)**	0.00 (NA to 0.629)	−55.9	0.0	**0.44 (0.34 to 0.53)**
		b			**0.45 (0.38 to 0.53)**				**0.45 (0.38 to 0.53)**
Total intestine	SVL	a	−23.2	19.8	0.01 (‐0.26 to 0.27)	0.69 (NA to 0.92)	−35.1	20.8	−0.16 (−0.51 to 0.19)
		b			**1.28 (0.93 to 1.63)**				**1.4 (1.01 to 1.73)**

Abbreviations: BM, body mass; SVL, snout‐vent length.

**Figure 3:**
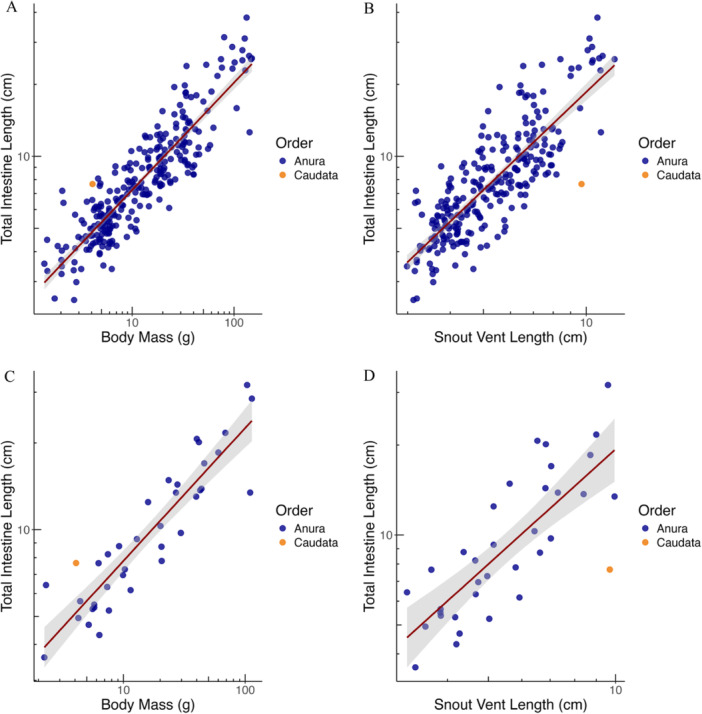
Intestine length relationships in amphibians (A) of all individuals with body mass (BM) (*n* = 423) and (B) all individuals with snout‐vent‐length (SVL) (*n* = 390), (C) species averages with BM (*n* = 38 species) and (D) species averages with snout‐vent length (*n* = 35 species). In some cases, the original data was presented as an average of several individuals; thus, in Figures A and B some points might represent more than one individual. Regression lines from GLS analyses. For statistics, see Table 1.

TIL scaled at a higher‐than‐geometric exponent to BM (95%CI of the scaling exponent well above 0.33); the geometric exponent of 1.0 was still included in the scaling of TIL with SVL in the GLS model, but not in the PGLS model (Table 1 and Figure 3). Furthermore, in the consistent data set, TIL was more tightly related to BM than to SVL (ΔAICc, Table 2), supporting the hyper‐allometry of TIL with body size.

**Figure 4:**
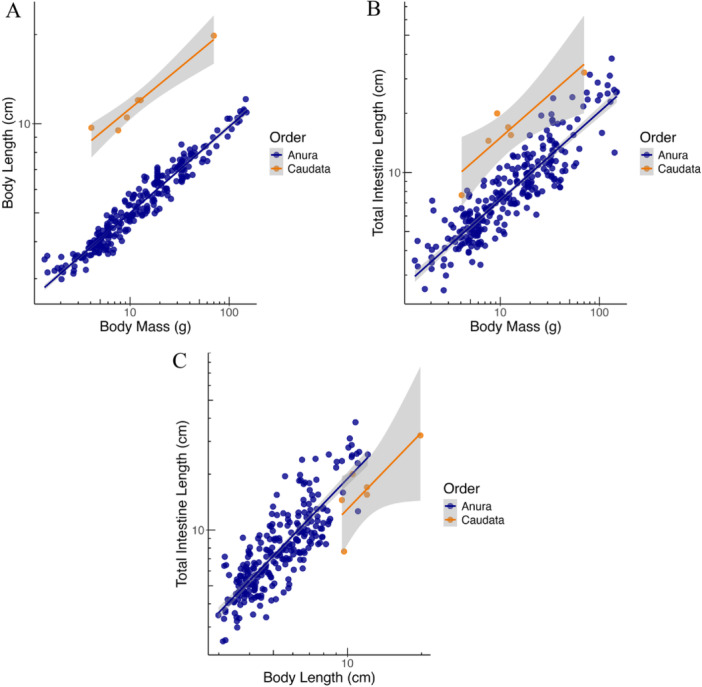
Allometric comparison between anurans (419 individuals) and caudata (6 individuals). Scaling of (A) snout‐vent‐length (SVL) with body mass (BM), (B) intestine length with BM, and (C) intestine length with SVL. Additional caudata data taken from (Pryor et al. 2006). Regression lines from GLS analyses.

TIL relative to BM was longer for Caudata compared to anurans (Figure 4A,B). On the other hand, Caudata intestine length was somewhat shorter for the same BL compared to anurans of the same length (Figure 4B,D); notably, this effect would possibly be more pronounced if the data for the greater siren individuals would not include the stomach (Pryor et al. 2006).

**Figure 5:**
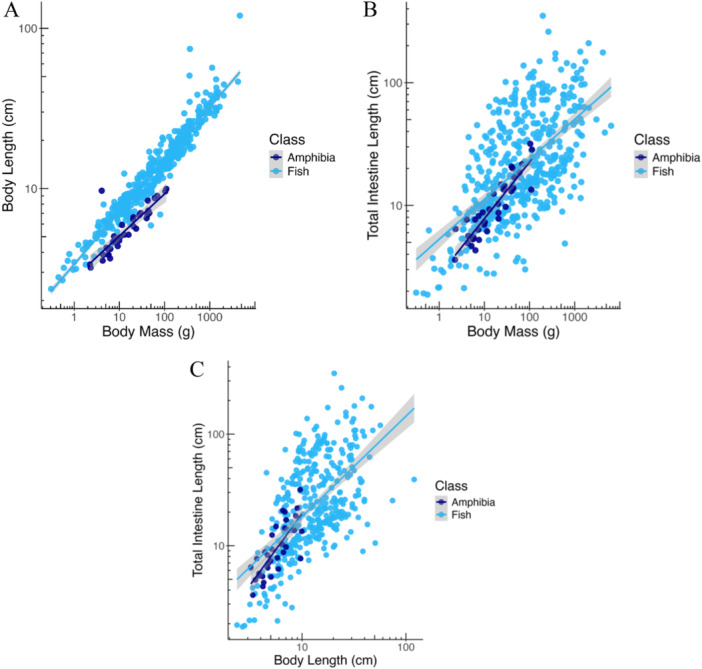
Comparison between amphibians and fishes: (A) body length (standard length in fish, snout‐vent‐length in amphibians) scaling with body mass (BM); (B) intestine length scaling with BM, and (C) intestine length scaling with body length. Fish data from (Duque‐Correa et al. 2024). Regression lines from GLS analyses.

**Figure 6:**
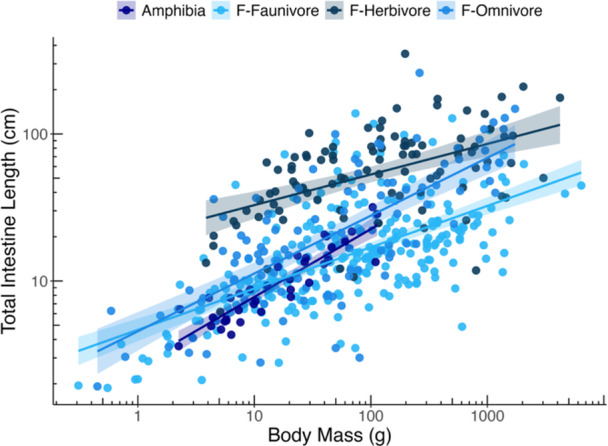
Allometric comparison between the intestine length—body mass scaling between amphibians and fishes of different trophic groups. Fish data from (Duque‐Correa et al. 2024). Regression lines from GLS analyses.

Anuran amphibians have a shorter body than fish at the same BM (Figure 5A). By contrast, relative to body size or BL, amphibian TIL is within the lower range of that of fishes (Figure 5B,C). When making the comparison more detailed by dividing the fishes into trophic categories, amphibian TIL is similar to that of faunivorous and omnivorous fishes and shorter than that of herbivorous ones (Figure 6).

## 4 Discussion

The absence of a significant phylogenetic signal in relationships of intestine length with either BM or BL indicates homogeneity in the relative intestine length across taxa, supporting the impression that adult amphibian digestive tracts show little interspecific differentiation (Stevens and Hume 1995). This aligns with the general observation that amphibians are comparatively homogenous in their trophic behavior. Notably, the present study did not comprise amphibian species whose adult forms are considered particularly herbivorous (Das 1996, Isaacs and Hoyos 2010, Hill et al. 2015, de‐Oliveira‐Nogueira et al. 2023). Therefore, whereas for most vertebrate clades, diet has been demonstrated to be associated with intestinal length (Wagner et al. 2009, Duque‐Correa et al. 2021, Hoppe et al. 2021, Duque‐Correa et al. 2022, Duque‐Correa et al. 2024), this is currently not possible in amphibians.

The results of our study underline the fundamental difference in the scaling of BL and TIL with BM. As found previously, BL in vertebrates typically scales very closely to the geometric (isometric) expectation with an exponent around BM^0.33^, or, vice versa, that BM scales with BL^3.00^ (Niklas 1994, Silva 1998, Duque‐Correa et al. 2024). This was also specifically demonstrated in various amphibian clades (Santini et al. 2018); that study also documented the evident fact that caudates generally have longer bodies than anurans of similar mass.

This scaling supports the hypothesis that organismal design roughly follows geometric principles, especially when assessed across a large variety of organisms at the whole‐body level. Nevertheless, individual body parts or organs may not necessarily follow this pattern, as for example illustrated for mammalian limb bones (Christiansen 1999). The intestinal tract represents another organ that does not scale isometrically with BM or BL; rather, it is hyper‐allometric, with a scaling exponent generally higher than those expected from sheer geometry. Our present study adds amphibians, nearly exclusively represented by anurans, as another vertebrate group in which this pattern is observed.

A plausible explanation for this intestine‐specific pattern was already mentioned in the Introduction. The tubular, “hose‐like” shape of the intestine is conveniently explained by the combined requirements of a large volume (i.e., a long tract) and short distances to the sites of digestive enzyme secretion and nutrient absorption. Under the assumption that for an efficient digestion, it is the latter that should not increase proportionately with body size, it would be understandable that the requirement for a larger organ at larger size is best met by an elongation of the tube, not by a proportionate increase in its diameter (Woodall and Skinner 1993).

Data to test this hypothesis are rare, and the present investigation is no exception. In the dissections performed for this study, no measure of intestinal diameter was taken due to the lack of an approach that could be reliably replicated across different observers (and due to the lack of literature data). When documenting dissections with photographs containing a scale, length can be reliably estimated, but this appears less reliable for an estimate of intestine “height”, from which a diameter could be deducted. To our knowledge, this relationship was only tested twice in multi‐species vertebrate studies. In fish, where the largest data collection indicated hyper‐allometric intestinal length scaling, a smaller data set (from Ghilardi et al. 2021) that contained both length and diameter data did not indicate a deviation from geometric expectations for either measurement (Duque‐Correa et al. 2024). By contrast, in a study on a limited number of ruminant species, Woodall and Skinner (1993) found that for the small intestine, length scaled with body mass hyper‐allometrically (exponent > 0.33) and diameter scaled with body mass hypo‐allometrically (exponent < 0.33). To our knowledge, these authors were the first to present the functional hypothesis also used as the explanation for the observed intestinal length scaling in the present study.

Hoppe et al. (2021) suggested that processes of body elongation might be linked to different embryological mechanisms between mammals and other vertebrates; this was based on the observation that mammals of an elongated body shape—the mustelids—appear to have particularly long intestinal tracts (though generally being considered faunivores) (McGrosky et al. 2016). This contrasts with elongated reptiles (snakes) (Hoppe et al. 2021), fish (e.g., moray) (Duque‐Correa et al. 2024) or amphibia (the salamander and sirenids of the present study), whose intestine appears short for their BL. Detailed studies on ontogenetic changes and the underlying regulators would be required to explore the mechanisms behind these differences.

The typical limitations to similar comparative studies apply, in particular with respect to the influence of body condition on the body measurements. Length measurements taken in different ways, such as by physical means (e.g., by caliper, Liao et al. 2016) or digitally (as in our own dissections) should be comparable and useful for evaluations across taxa; however, when comparing two specific species, data taken under exactly the same methods should be preferred. Evidently, a larger range of non‐anuran species, including not only caudata but also caecilians, would be welcome to corroborate the generality of our findings that are based mainly on anurans. Zoological collections maintaining such species might be a good source for specimens in the future. Data on tadpole stages, though existing to some degree, were not included, and a specific evaluation of the intestinal anatomy of amphibian tadpoles of various developmental stages and trophic behaviors might be of interest.

A standard expectation with relation to the digestive tract is that it corresponds, in its anatomy, to the trophic level of the organism (Chivers and Hladik 1980, Stevens and Hume 1998, Karasov et al. 2011, Langer and Clauss 2018). Quantitative evaluation has supported this assumption statistically, with herbivorous animals typically having longer intestines than faunivores. But the scatter in these relationships is remarkable, making it necessary to emphasize that while this is often a statistically valid result, there is apparently a large degree of flexibility in terms of resources that can be used with a given anatomy, and additional factors might explain the observed patterns (Duque‐Correa et al. 2021, Hoppe et al. 2021, Duque‐Correa et al. 2022, Duque‐Correa et al. 2024). For amphibia, the demonstrated relationship between brain size and intestine length, congruent with the expensive tissue hypothesis (Liao et al. 2016), offers one possible additional explanation for variation in the intestine‐body mass relationship. Nevertheless, in a larger framework, for the amphibian species investigated in the present study, the magnitude of their intestinal lengths is similar to that of faunivorous fish, supporting the assumption that faunivores do not need particularly long intestines.

## Author Contributions

**M. J. Duque‐Correa:** conceptualization, investigation, writing – original draft, methodology, data curation, formal analysis, visualization, project administration. **C. Meloro:** writing – review and editing, investigation, methodology. **S. Keller:** writing – review and editing, investigation. **P. Cigler:** writing – review and editing, investigation. **I. Wethli:** writing – review and editing, investigation. **J. Niehaus:** writing – review and editing, investigation. **M. Przybyło:** writing – review and editing, investigation. **M. Clauss:** conceptualization, writing – original draft, validation, supervision, methodology.

## Conflicts of Interest

The authors declare no conflicts of interest.

## Supporting information

Supporting File

## Data Availability

All data used in this study are provided as supporting materials.
